# Supplementary figures and images for: Genetic structure and molecular diversity of Brazilian grapevine germplasm: Management and use in breeding programs
Source: PLoS One. 2020 Oct 15;15(10):e0240665. doi: 10.1371/journal.pone.0240665 (PMC7561202; doi:10.1371/journal.pone.0240665)

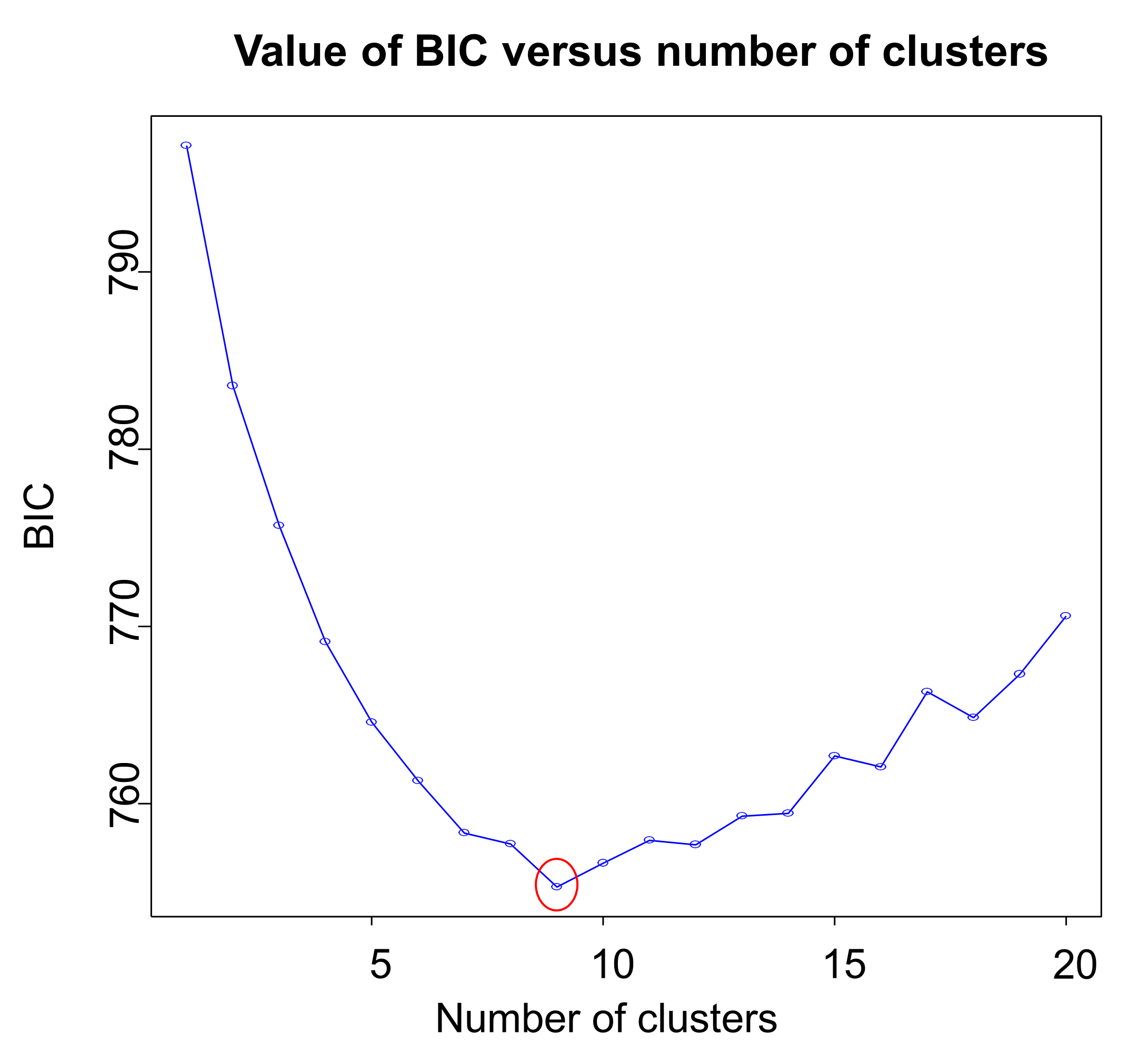

Supplement: S1 Fig — The accepted true number of clusters was nine. (TIF) [file pone.0240665.s004.tif]

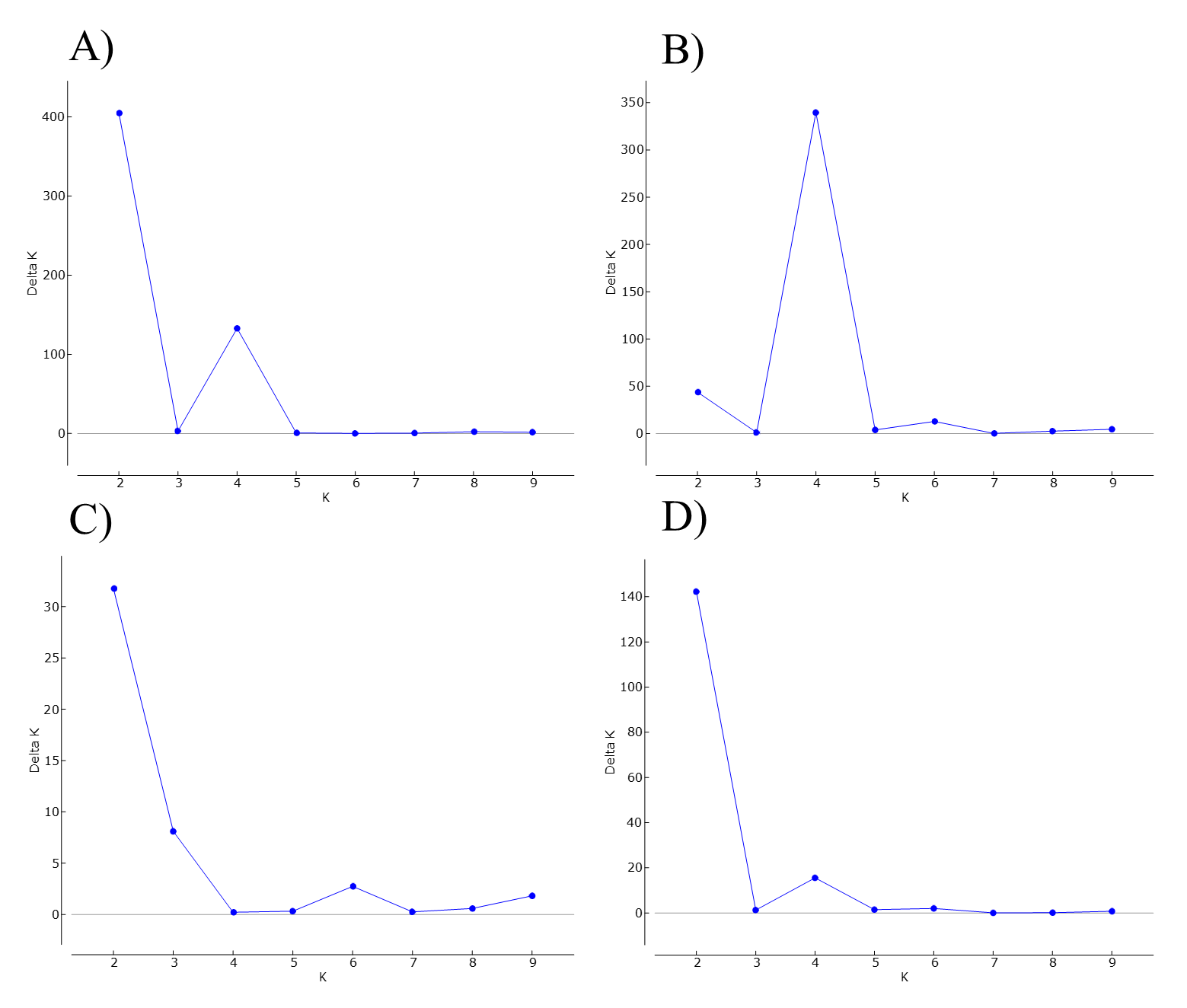

Supplement: S2 Fig — Graphics for the detection of the most probable number of groups (K) estimated based on the method described by Evanno et al. (2005) [51]. (A) Cluster 1—Highest peak for K = 2. (B) Cluster 2—Highest peak for K = 4. (C) Cluster 3—Highest peak for K = 2. (D) Admixture group—Highest peak for K = 2. (TIF) [file pone.0240665.s005.tif]
